# Anaplastic Transformation of Sphenoid Wing Meningioma With Orbital and Cavernous Sinus Invasion: Unveiling the Aggressive Nature

**DOI:** 10.7759/cureus.57025

**Published:** 2024-03-27

**Authors:** Plamen Penchev, Borislav Kalnev, Stela Petrova, Petar-Preslav Petrov, Mihail Kalnev

**Affiliations:** 1 Faculty of Medicine, Medical University of Plovdiv, Plovdiv, BGR; 2 Department of Neurological Surgery, Medical University of Plovdiv, Plovdiv, BGR; 3 Clinic of Neurological Surgery, University Multi-profile Hospital for Active Treatment (UMHAT) Saint George, Plovdiv, BGR; 4 Department of General and Clinical Pathology, University Multi-profile Hospital for Active Treatment and Emergency Medicine (UMHATEM) Pirogov, Sofia, BGR; 5 Department of Anatomy, Histology and Embryology, Medical University of Plovdiv, Plovdiv, BGR

**Keywords:** case report, optic nerve, orbit, cavernous sinus, sphenoid bone, meningioma, anaplastic meningioma

## Abstract

Primary tumors in the central nervous system, known as meningiomas, are frequently found and constitute a substantial proportion of tumor cases. Although generally benign, there are occasional cases where they might exhibit malignant characteristics. Anaplastic meningioma is a rare subtype of malignant meningiomas, representing only a small proportion of cases. We present the case of a 70-year-old female patient who presented to the Neurosurgery Clinic of University Hospital “Saint George” with clinical manifestations of monocular vision and blurry vision in the right eye for three months. On physical examination, unilateral ptosis and mydriasis were noted in the left eye. MRI revealed an extra-axial mass located supratentorial in the left temporopolar region affecting the wing of the left sphenoidal bone, invading the cavernous sinus, suppressing the left and right optic nerves, and involving the left orbit. Operative treatment was performed through a left pterional craniotomy and resection of the tumor mass by microsurgical technique. The subdural, epidural, and intraorbital mass were resected. Total removal of the tumor was not achievable and subtotal resection was performed. Pathology results showed that the tumor mass was anaplastic meningioma. Surgery-related complications were not observed. Postoperatively, the patient was mobilized on the day after intervention and the control CT scan showed no ischemic or hemorrhagic events. The patient experienced relief in her symptoms and was discharged on the fifth day. The patient underwent radiation therapy, resulting in the complete removal of the left tumor in the cavernous sinus. After six months, no tumor recurrence was found, and a long-term follow-up is planned to monitor for possible recurrence.

## Introduction

Anaplastic meningioma (AM) is a malignant tumor of significant aggressiveness that has been categorized as a grade 3 meningioma based on the 2021 categorization of tumors affecting the central nervous system by the World Health Organization (WHO) [1]. Despite its high recurrence rate and unfavorable prognosis, AM is a rare subtype, constituting around 1%-2% of all meningiomas [2,3]. CT scans and MRIs are typically effective in diagnosing typical cases of AM. However, when AM infiltrates the adjacent structures and induces concomitant alterations, the diagnosis becomes more challenging with a risk of misdiagnosis [4].

This case report aims to emphasize the diagnostic difficulties associated with sphenoid wing meningiomas that have benign radiological features on MRI, yet display invasive behavior in important anatomical structures such as the cavernous sinus and orbit. This report seeks to emphasize the significance of conducting a thorough diagnostic assessment and taking into account the aggressive histopathological characteristics, even in tumors that were initially considered benign based on radiological appearance, by providing a detailed account of the intraoperative findings and subsequent pathology results that revealed the presence of AM.

## Case presentation

**Figure 1:**
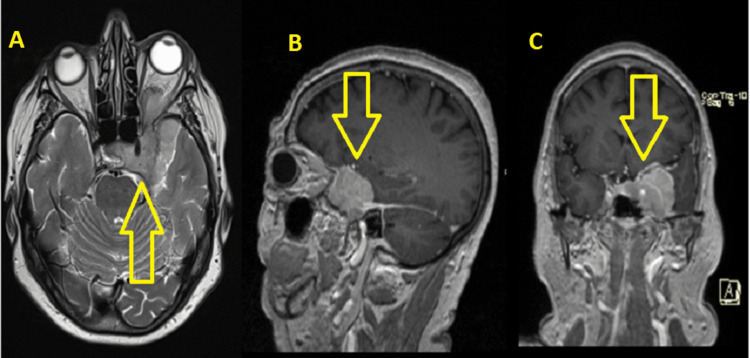
Preoperative MRI. Radiological findings of the tumor. A - Axial plane. B - Sagittal plane. C - Coronal plane.

We present the case of a 70-year-old female patient who presented to the Neurosurgery Clinic of University Hospital “Saint George” with clinical manifestations of monocular vision and blurry vision in the right eye for three months. On physical examination, unilateral ptosis and mydriasis were noted in the left eye. MRI revealed a hyperintense extra-axial mass located supratentorial in the left temporopolar region affecting the wing of the left sphenoidal bone, invading the cavernous sinus, suppressing the left and right optic nerves, and involving the left orbit, suspicious for sphenoid wing meningioma (Figure 1).

**Figure 2:**
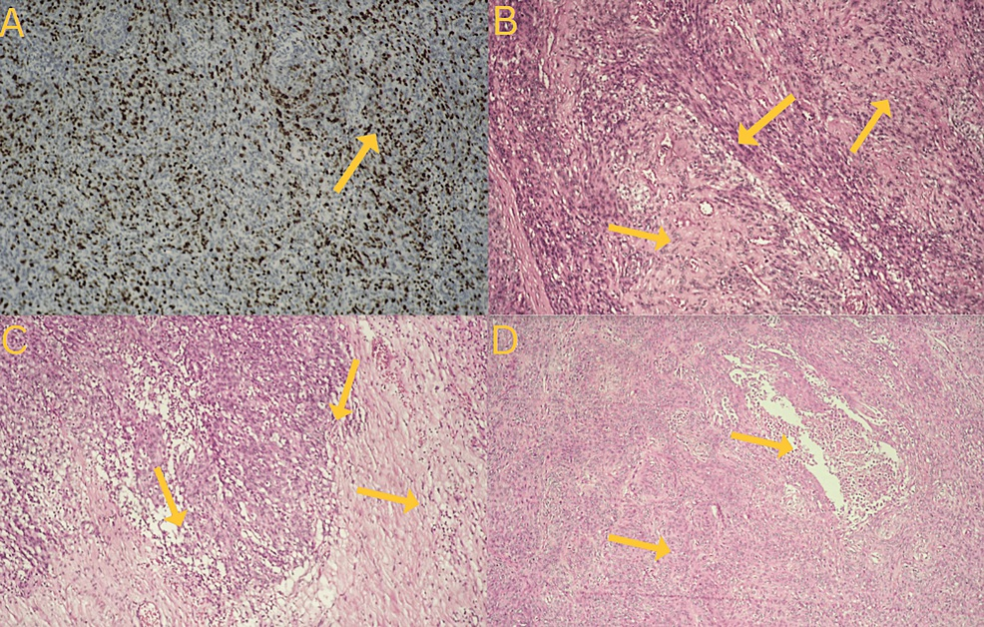
Histological results. Anaplastic meningioma. A -  An immunohistochemistry proliferation marker test using KI-67 revealing elevated mitotic index and mitotic count above 20 mitoses per 10 consecutive high-power fields. Brain invasion (B). B, C, D - Nested structure, hypercellular regions, numerous mitoses, and nuclear inclusions are seen (hematoxylin and eosin staining).

**Figure 3:**
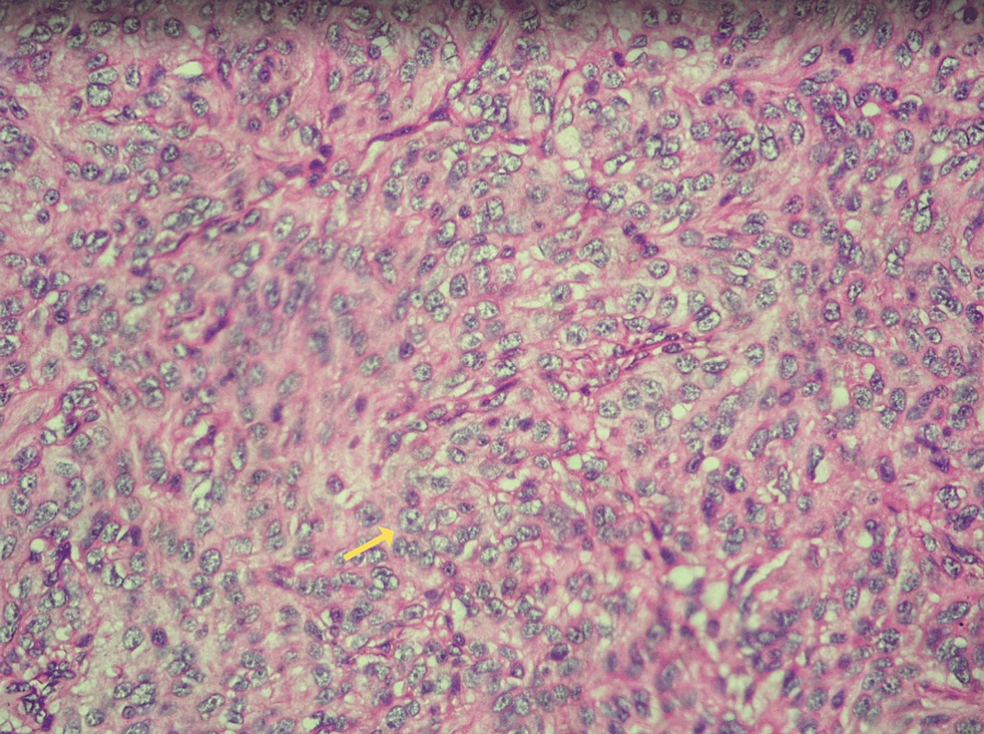
Histology results. Anaplastic meningioma. Increased mitotic activity - the presence of irregular mitoses and prominent nucleoli.

Under general anesthesia, operative treatment was performed through a left pterional craniotomy and resection of the tumor mass by microsurgical technique under ultrasound control. The subdural, epidural, and intraorbital mass were resected. Total removal of the tumor was not achievable and subtotal resection was performed. Pathological results showed that the tumor mass was AM, a meningioma with clearly malignant cytomorphology that may resemble carcinoma, melanoma, or high-grade sarcoma on the histologic pattern. The tumor showed characteristics of AM with a varied structure ranging from well-differentiated meningioma-like sections to sarcoma-like sections and invasive growth toward the brain substance (Figure 2B). The features of cellular and nuclear atypia were present and necrosis and brain invasion were noted accessible. An immunohistochemistry proliferation marker test using Ki-67 was conducted, revealing an elevated mitotic index and mitotic count above 20 mitoses per 10 consecutive high-power fields (Figure 2A). Additionally, nested structure, hypercellular regions, numerous mitoses, and nuclear inclusions were seen (Figures 2B-2D), along with brain invasion (Figure 2B). Increased mitotic activity was noted with the presence of irregular mitoses and prominent nucleoli (Figure 3). Occasionally, histologic or immunohistochemical evidence of meningothelial whorls, psammosomal bodies, or nuclear pseudoinclusions can be noted. We did not investigate other immunohistochemistry markers as we considered them unnecessary, given the diagnosis was based on just morphology.

**Figure 4:**
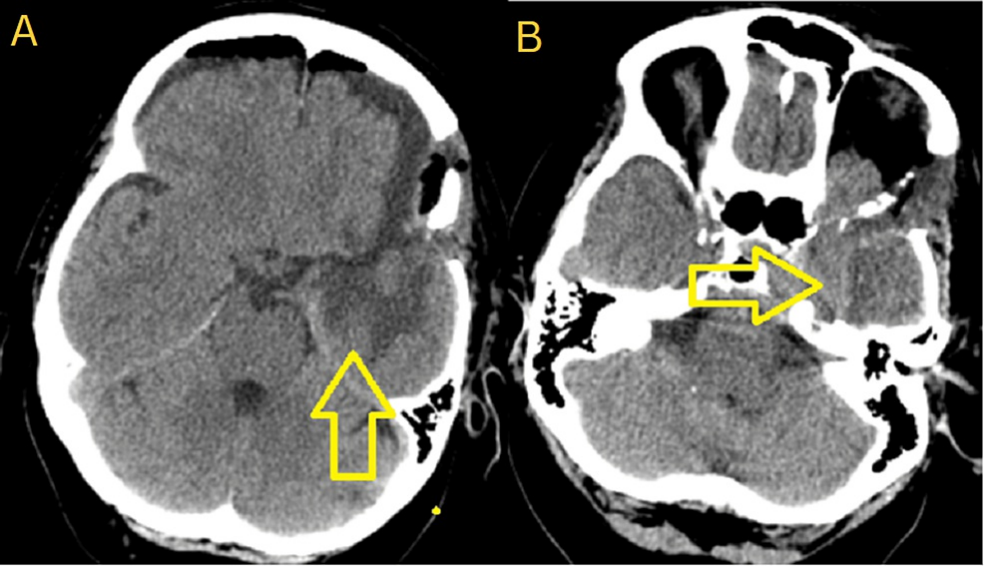
Postoperative CT scan. А - Аxial plane. Subtotal resection. No evidence of ischemic or hemorrhagic events. B - Axial plane. Subtotal resection. No evidence of ischemic or hemorrhagic events.

Surgery-related complications were not observed. Postoperatively, the patient was mobilized on the day after intervention and the control CT scan showed no ischemic or hemorrhagic events (Figure 4).

Following the surgical procedure, the patient underwent radiation therapy to specifically target any remaining tumor cells and decrease the likelihood of a recurrence. Photon-based external-beam radiation therapy (EBRT) was chosen due to its precision in targeting intracranial tumors while minimizing damage to the surrounding tissue. Considering the subtotal resection and the aggressive behavior of the tumor, a dose escalation approach was employed. The radiation dosage administrated was between 60 and 66 Gy, delivered over a schedule of 30 to 33 sessions. The escalated dose aimed to improve tumor control and reduce the chance of recurrence. The patient responded very well to the treatment, resulting in the complete removal of the left tumor within the cavernous sinus. The patient reported symptomatic relief following treatment and no complications or worsening of symptoms following/during the therapy were observed. A control CT scan performed approximately six months after completion of radiation therapy showed no evidence of tumor recurrence in the treated area. The patient remained asymptomatic, indicating a favorable response to treatment. Long-term follow-up is planned to continue monitoring for any signs of tumor recurrence or progression.

## Discussion

Classification and incidence

The most prevalent intracranial brain tumor is meningioma, which accounts for more than one-third of primary brain neoplasms [1,2]. There are 15 histological subtypes and three grades of meningioma [3]. AM is the predominant grade 3 variant, characterized by significant aggressive behavior and a high risk of recurrence [1]. AM typically manifests between the ages of 18 and 70 years, as seen in our patient [4]. Progesterone hormone receptors are present in meningiomas, which are more prevalent in females. Atypical and anaplastic grades can be observed in both males and females [5].

Clinical manifestation

AM exhibits atypical clinical features, with the majority of patients displaying signs and symptoms associated with mass effects at the tumor site, such as headache, seizure, and hemiparesis. However, a subset of patients may remain asymptomatic [6]. Our patient exhibited distinctive symptoms, including visual impairment and unilateral vision.

Radiological characteristics

AM can display diverse manifestations on CT and MRI, although it may also possess certain similar characteristics. The majority of AM cases exhibit a broad tumor base and typically possess lobulated or irregular fusiform morphology, as observed in the current case. AM is typically observed as a low-density area on a routine CT scan with areas of cystic degeneration and calcification seen in the shadow. AM can be found in several anatomical places, such as the convexity dura mater, skull base, tentorium, falx, or intraventricular region [7].

In this case, the tumor had radiological features consistent with benign sphenoid wing meningioma. However, the pathological findings substantiated its malignant nature. Therefore, there was an unconventional tumor bed. The tumor exhibits iso- to hypointensity on T1-weighted imaging and iso- to hyperintensity on T2-weighted imaging on conventional MRI [8]. In our case, the MRI revealed a hyperintensity tumor lesion on T2-weighted imaging that extensively invaded the orbit and cavernous sinus while also compressing the left optic nerve.

Histopathology

The identification of AM depends on the observation of morphological features and the presence of immunohistochemical markers that are expressed inside the tumor cells such as progesterone receptor (PR) in the membrane and epithelial membrane antigen and Somatostatin receptor 2a in the cytoplasm. *TERT *promoter mutation and *CDKN2A/B* homozygous deletion are defying mutations for Grade 3. Besides, the loss of trimethylation (H3K27me3 loss) is also seen in aggressive cases with poor overall survival. The AM gross specimen exhibits a grayish-red coloration. Tumor microscopy displays either malignant cytology, characterized by the presence of carcinoma, sarcoma, or melanoma-like histology, or a significantly increased mitotic index, and a count of at least 20 mitoses per 10 consecutive high-power fields. Additionally, the tumor has >20 mitotic figures per 10 consecutive high-power fields [9,10].

The pathology findings in our patient verified the presence of necrosis, de-differentiated sections resembling meningiomas to sarcomas. A Ki-67 proliferation marker test was performed, which showed an increased mitotic index and mitotic count exceeding 20 mitoses per 10 consecutive high-power fields. There is a correlation between the findings of the aforementioned investigations. We did not investigate other immunohistochemistry markers as we considered them unnecessary, given the diagnosis was based on just morphology.

Management of anaplastic meningiomas

While surgery is widely regarded as the major therapeutic approach in such cases, the elevated rates of recurrence and metastasis necessitate the utilization of other adjuvant therapeutic approaches, such as external-beam radiation [6,7]. In our patient, we performed a subtotal resection to prevent harm to vital neurovascular structures in the cavernous sinus. The patient underwent radiation therapy to target the remaining tumor cells and reduce the risk of recurrence. Photon-based EBRT was chosen due to its precision for intracranial tumors and minimal tissue damage. The radiation dosages ranged from 60-66 Gy and were delivered over 30-33 sessions. The patient responded well, removing the left tumor within the cavernous sinus, and showed no worsening of symptoms or complications during/after the therapy. A six-month CT scan showed no evidence of tumor recurrence in the treated area. Long-term follow-up is planned to monitor for any signs of recurrence or progression.

The incidence of tumor regrowth is around 80%. However, the occurrence of extracranial metastases in these cases is rare, representing a mere 0.1% of cases. Surgical treatment is the only effective approach in the management of malignant meningiomas. In recent years, there have been notable advancements in surgical technique and a reevaluation of surgical anatomy, leading to the implementation of aggressive strategies in the treatment of brain tumors.

Notwithstanding the complete removal of the tumor, the survival rate for malignant meningiomas in the absence of adjuvant therapy is below two years [11,12]. Patients diagnosed with malignant meningiomas who underwent surgical intervention and received adjuvant therapy, such as radiation alone or radiation combined with chemotherapy, exhibited a median survival time of five years. Furthermore, the extent of tumor resection did not demonstrate any significant association with the likelihood of recurrence [13]. The efficacy of focalized EBRT or stereotactic radiosurgery in enhancing tumor control and survival rates among patients diagnosed with subtotal resected, recurring, and malignant meningiomas has been proven [14-17].

According to Goldsmith et al., the five-year progression-free survival rate following subtotal resection and radiation therapy was 89% for benign meningiomas and 48% for malignant meningiomas. The study also revealed that younger age and treatment with innovative technologies after 1980 were associated with an enhanced progression-free survival rate [18]. After six months of radiation therapy, our patient showed no evidence of recurrence. However, it is crucial to arrange a long-term follow-up for a minimum of five years.

## Conclusions

To summarize, this case emphasizes the misleading nature of sphenoid wing meningiomas, which might appear harmless on MRI but have aggressive histological markers such as aggressive histopathology. The disparity observed between the radiological presentation and the observations made during surgery highlights the importance of employing a complete diagnostic strategy that integrates sophisticated imaging techniques, intraoperative evaluation, and histological analysis.

Clinicians must uphold a heightened level of skepticism regarding atypical meningiomas, particularly when confronted with tumors exhibiting invasive qualities toward critical neurovascular structures. Prompt identification and precise diagnosis are vital for directing suitable treatment approaches and maximizing patient outcomes. The need for additional research and heightened awareness is justified to enhance diagnostic criteria and prognostic classification of these complex tumors.

## References

[1] Torp SH, Solheim O, Skjulsvik AJ (2022). The WHO 2021 classification of central nervous system tumours: a practical update on what neurosurgeons need to know-a minireview. Acta Neurochir (Wien).

[2] Orton A, Frandsen J, Jensen R, Shrieve DC, Suneja G (2018). Anaplastic meningioma: an analysis of the National Cancer Database from 2004 to 2012. J Neurosurg.

[3] Ostrom QT, Gittleman H, Farah P (2013). CBTRUS statistical report: primary brain and central nervous system tumors diagnosed in the United States in 2006-2010. Neuro Oncol.

[4] Aizer AA, Bi WL, Kandola MS (2015). Extent of resection and overall survival for patients with atypical and malignant meningioma. Cancer.

[5] Cornelius JF, Slotty PJ, Steiger HJ, Hänggi D, Polivka M, George B (2013). Malignant potential of skull base versus non-skull base meningiomas: clinical series of 1,663 cases. Acta Neurochir (Wien).

[6] Sughrue ME, Sanai N, Shangari G, Parsa AT, Berger MS, McDermott MW (2010). Outcome and survival following primary and repeat surgery for World Health Organization Grade III meningiomas. J Neurosurg.

[7] Durand A, Labrousse F, Jouvet A (2009). WHO grade II and III meningiomas: a study of prognostic factors. J Neurooncol.

[8] Kane AJ, Sughrue ME, Rutkowski MJ (2011). Anatomic location is a risk factor for atypical and malignant meningiomas. Cancer.

[9] Hanft S, Canoll P, Bruce JN (2010). A review of malignant meningiomas: diagnosis, characteristics, and treatment. J Neurooncol.

[10] Cimino PJ (2015). Malignant progression to anaplastic meningioma: neuropathology, molecular pathology, and experimental models. Exp Mol Pathol.

[11] Thomas HG, Dolman CL, Berry K (1981). Malignant meningioma: clinical and pathological features. J Neurosurg.

[12] Younis GA, Sawaya R, DeMonte F, Hess KR, Albrecht S, Bruner JM (1995). Aggressive meningeal tumors: review of a series. J Neurosurg.

[13] Dziuk TW, Woo S, Butler EB (1998). Malignant meningioma: an indication for initial aggressive surgery and adjuvant radiotherapy. J Neurooncol.

[14] Falavigna A, Santos JA, Chimelli L, Ferraz FA, Bonatelli Ad Ade P (2001). Anaplastic meningioma: case report. Arq Neuropsiquiatr.

[15] Kondziolka D, Lunsford LD, Coffey RJ, Flickinger JC (1991). Stereotactic radiosurgery of meningiomas. J Neurosurg.

[16] Maire JP, Caudry M, Guérin J (1995). Fractionated radiation therapy in the treatment of intracranial meningiomas: local control, functional efficacy, and tolerance in 91 patients. Int J Radiat Oncol Biol Phys.

[17] Chamberlain MC (1996). Adjuvant combined modality therapy for malignant meningiomas. J Neurosurg.

[18] Goldsmith BJ, Wara WM, Wilson CB, Larson DA (1994). Postoperative irradiation for subtotally resected meningiomas. A retrospective analysis of 140 patients treated from 1967 to 1990. J Neurosurg.

